# Perioperative cerebral ischemia promote infiltrative recurrence in glioblastoma

**DOI:** 10.18632/oncotarget.3994

**Published:** 2015-05-04

**Authors:** Anna-Luisa Thiepold, Sebastian Luger, Marlies Wagner, Natalie Filmann, Michael W. Ronellenfitsch, Patrick N. Harter, Anne K. Braczynski, Stephan Dützmann, Elke Hattingen, Joachim P. Steinbach, Christian Senft, Johannes Rieger, Oliver Bähr

**Affiliations:** ^1^ Dr. Senckenberg Institute of Neurooncology, Goethe-University Hospital, Frankfurt, Germany; ^2^ Department of Neurology, Goethe-University Hospital, Frankfurt, Germany; ^3^ Institute of Neuroradiology, Goethe-University Hospital, Frankfurt, Germany; ^4^ Institute of Biostatistics and Mathematical Modeling, Goethe-University Hospital, Frankfurt, Germany; ^5^ Edinger Institute, Institute of Neurology, Goethe-University Hospital, Frankfurt, Germany; ^6^ Department of Neurosurgery, Goethe-University Hospital, Frankfurt, Germany

**Keywords:** glioblastoma, hypoxia, patterns of progression, perioperative ischemia, MRI

## Abstract

**Background:**

Hypoxia is a key driver for infiltrative growth in experimental gliomas. It has remained elusive whether tumor hypoxia in glioblastoma patients contributes to distant or diffuse recurrences. We therefore investigated the influence of perioperative cerebral ischemia on patterns of progression in glioblastoma patients.

**Methods:**

We retrospectively screened MRI scans of 245 patients with newly diagnosed glioblastoma undergoing resection for perioperative ischemia near the resection cavity. 46 showed relevant ischemia nearby the resection cavity. A control cohort without perioperative ischemia was generated by a 1:1 matching using an algorithm based on gender, age and adjuvant treatment. Both cohorts were analyzed for patterns of progression by a blinded neuroradiologist.

**Results:**

The percentage of diffuse or distant recurrences at first relapse was significantly higher in the cohort with perioperative ischemia (61.1%) compared to the control cohort (19.4%). The results of the control cohort matched well with historical data. The change in patterns of progression was not associated with a difference in survival.

**Conclusions:**

This study reveals an unrecognized association of perioperative cerebral ischemia with distant or diffuse recurrence in glioblastoma. It is the first clinical study supporting the concept that hypoxia is a key driver of infiltrative tumor growth in glioblastoma patients.

## INTRODUCTION

The course of disease of patients with glioblastoma is almost universally progressive and ultimately fatal. At first recurrence, local re-growth is the most frequent pattern of progression with approximately 80%, whereas diffuse or distant progression is rare. Even at later recurrences local tumor growth remains the predominant pattern with about 71%.[1] Nevertheless, the diffusely infiltrative pattern of glioblastoma growth is one of the most difficult obstacles for next-generation glioblastoma therapies aiming at long-term disease control.

Hypoxia has a fundamental role in the microenvironment of glioblastoma and is known to promote resistance to radio- and chemotherapy. Moreover, hypoxia is a key driver for invasive growth and distant metastasis.[2-5] Mechanisms include the detection of hypoxia by hypoxia-inducible-factor (HIF)-1-alpha signaling and the generation of adaptive responses including enhanced migration and invasion that may be mediated by c-MET and other factors.[6-8] That hypoxia is a key driver for invasive glioma growth has been demonstrated in preclinical studies *in vitro* and *in vivo*. However, evidence that this is true in glioma patients is missing. In the current study we took advantage of the fact that some glioma patients suffer a cerebral ischemia during resection and thereby presumably exposing residual tumor cells to hypoxia. While antiangiogenic therapies have been extensively studied in glioblastoma, the time course and degree of hypoxia induced by these remain controversial.[9-14] In contrast, extent and time course of cerebral hypoxia caused by ischemic stroke can be much better defined. Since cerebral ischemia can be a complication following tumor resection, mainly by arterial or venous vessel injury during surgery,[15] we performed a retrospective study addressing the association of postoperative ischemia with patterns of recurrence in glioblastoma patients. We hypothesized that perioperative ischemia near the resection cavity might induce a more invasive pattern of recurrence by induction of sublethal hypoxia in residual tumor cells. We therefore screened our local brain tumor database for patients with relevant lesions of restricted diffusion on immediate postoperative MRI. The patterns of recurrence in these patients were analyzed by a blinded neuroradiologist. The results were compared with a cohort of matched pairs of patients without perioperative stroke and historical data.

## RESULTS

### Patient characteristics

Patient characteristics were representative for a cohort of glioblastoma patients undergoing resection (Table 1). All patients with perioperative ischemia had a volume of restricted diffusion of at least 3 cm³. Median volume of restricted diffusion in this cohort was 17.1 cm³ (average 22.3 cm³, range 3.4-61.5 cm³).

**Table 1:** Patient characteristics

Characteristics	Patients with perioperative ischemia (n=46)	Matched pairs without perioperative ischemia (n=46)
General		
Age, median (range)	62 (25-79)	63 (36-79)
Female, (n)	39.1% (18)	39.1% (18)
KPS, median (range)	80% (20-100)	90% (40-100)
Surgery		
GTR or STR, (n)	84.8% (39)	82.6% (38)
Partial resection, (n)	15.2% (7)	15.2% (7)
Unknown	0% (0)	2.2% (1)
Histology		
Glioblastoma, (n)	100% (46)	100% (46)
MGMT-Promoter		
Methylated	45.7% (21)	41.3% (19)
Not methylated	45.7% (21)	47.8% (22)
Unknown	8.7% (4)	10.9% (5)
Days to treatment, median (range)	28 (12-66)	30 (13-345)
Adjuvant treatment		
Radiochemotherapy, (n)	71.7% (33)	71.7% (33)
Radiotherapy, (n)	10.9% (5)	10.9 % (5)
Temozolomide, (n)	4.3% (2)	4.3% (2)
Unknown, (n)	10.9 % (5)	10.9% (5)
No therapy, (n)	2.2% (1)	2.2% (1)

Distribution of the baseline characteristics including age, Karnofsky performance score (KPS), type of resection, histology and MGMT methylation status were comparable between both groups. Since we also matched for adjuvant treatment until first recurrence these parameters were identical. In both groups, 71.7% of the patients were treated with radiotherapy plus concomitant and adjuvant temozolomide according to the EORTC 26981/22981-NCIC CE3 study.[16] In some older patients radiotherapy or temozolomide were administered alone.[17]

A longer period between resection and start of adjuvant treatment could be a reasonable factor influencing patterns of progression. Regarding this aspect, we did not find a difference between the both groups (Table 1).

In each group 10 patients were not fully evaluable and regarded as drop-outs. In the control cohort one patient has not progressed yet, 3 patients had insufficient radiology and 6 patients were lost for follow-up. In the ischemia cohort 2 patients had not progressed yet, 2 patients had deceased before progression and 6 patients were lost for follow-up.

**Figure 1:**
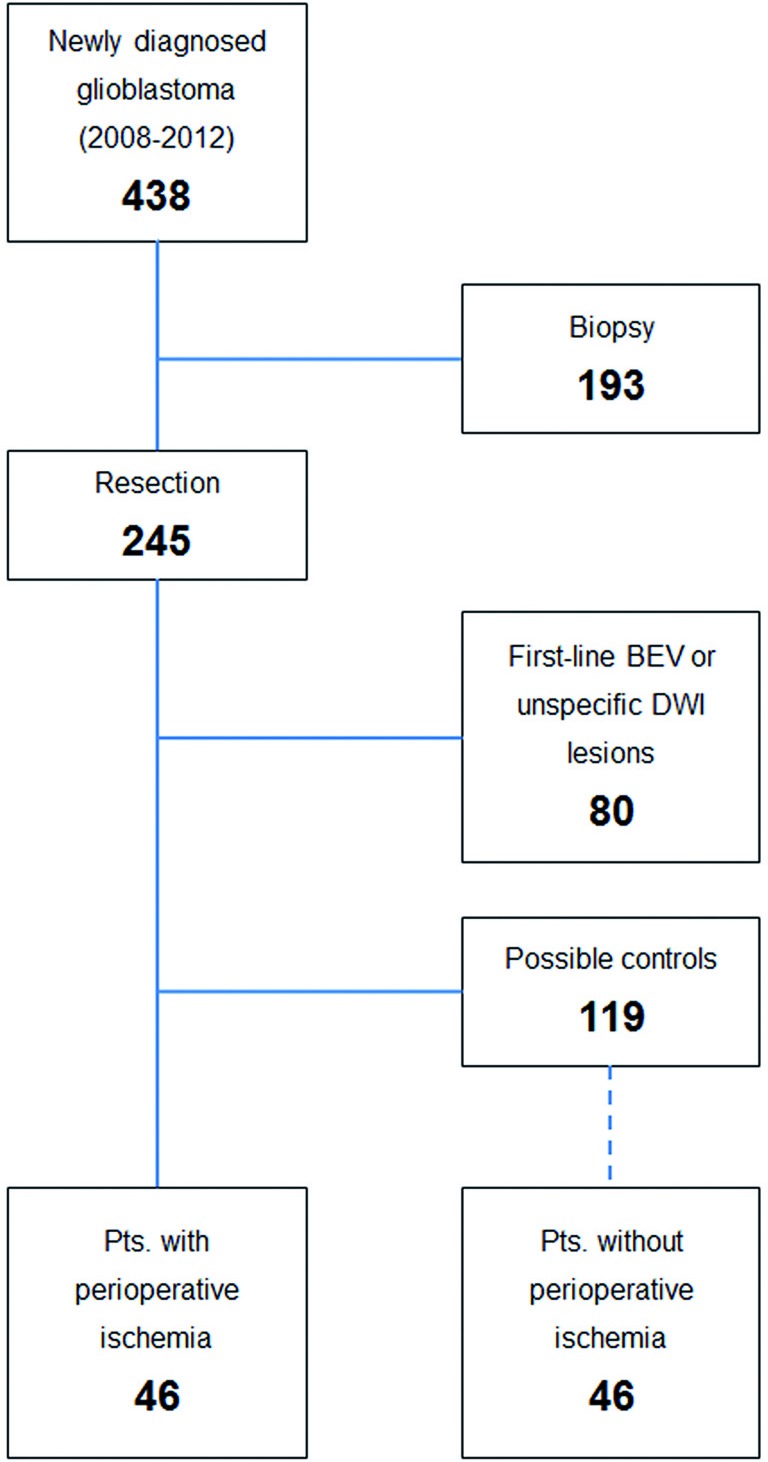
Patient selection Patient selection of the current study is shown. 193 patients were biopsied and therefore excluded. Another 80 patients were excluded due to small and unspecific lesions of restricted diffusion or BEV first line treatment. In 46 patients we identified unequivocal new perioperative cerebral ischemia on postoperative MRI, whereas 119 did not show lesions of restricted diffusion. These patients served as selection cohort for the 1:1 matched pairs analysis as described in methods.

**Figure 2:**
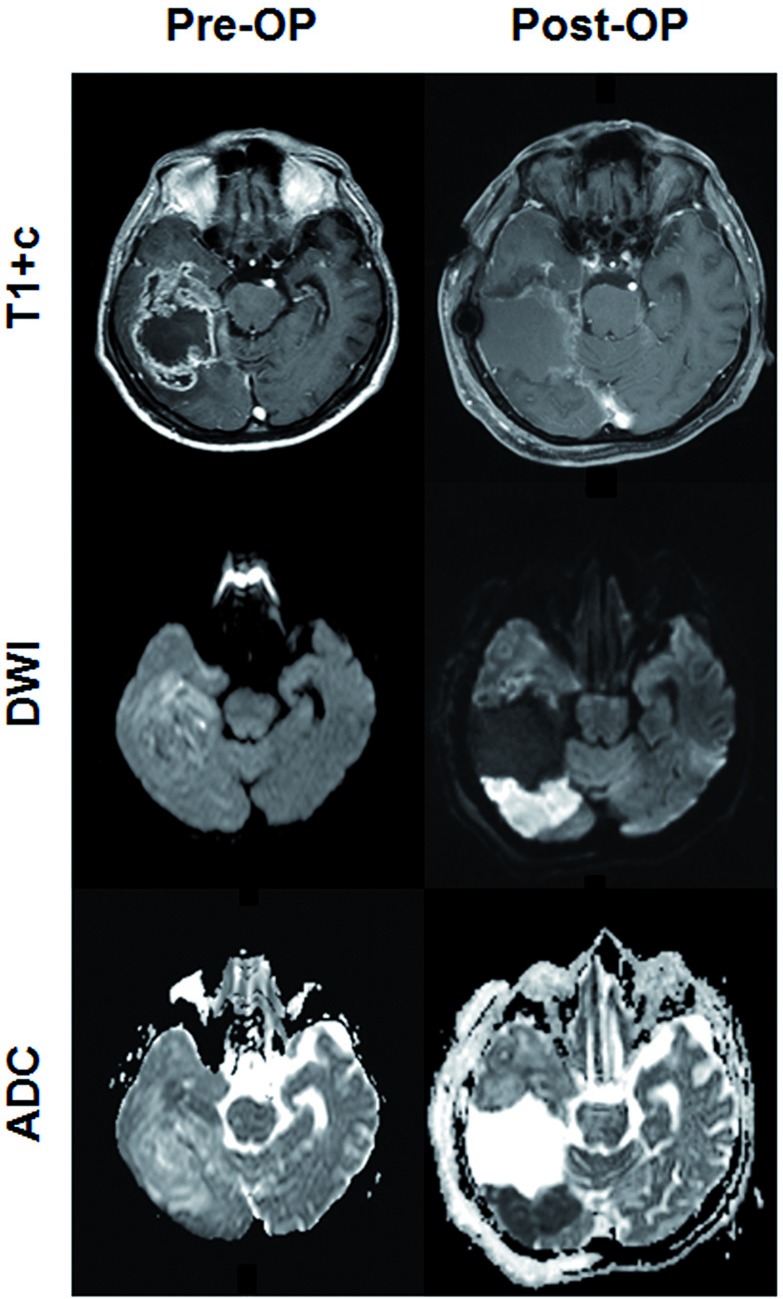
Perioperative ischemia Representative pre- and postoperative (< 72h after surgery) MRI scans from a patient with perioperative ischemia ARE shown.

**Figure 3:**
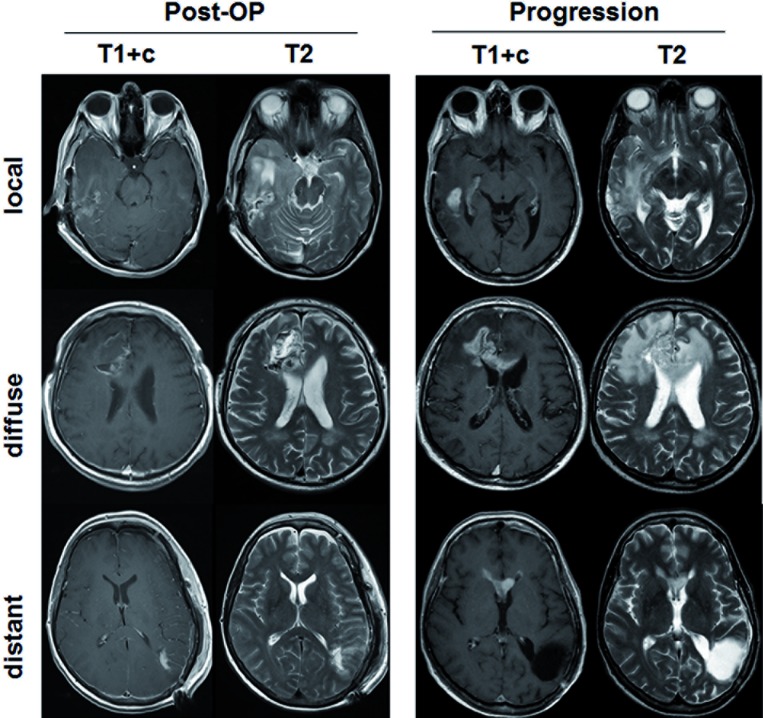
Represantative images for patterns of progression MRI scans for the three types of progression (local, diffuse, distant), as predefined in Methods, are shown. The first two columns show the postoperative situation on contrast enhanced T1 sequences (T1+c) and T2 weighted sequences. The last two columns show the corresponding scans at the time of first progression.

### Patterns of recurrence

In each group 36 patients had radiologically confirmed first recurrence and were fully available for analysis of pattern of progression. Recurrences did not involve the area of former ischemia.

**Figure 4:**
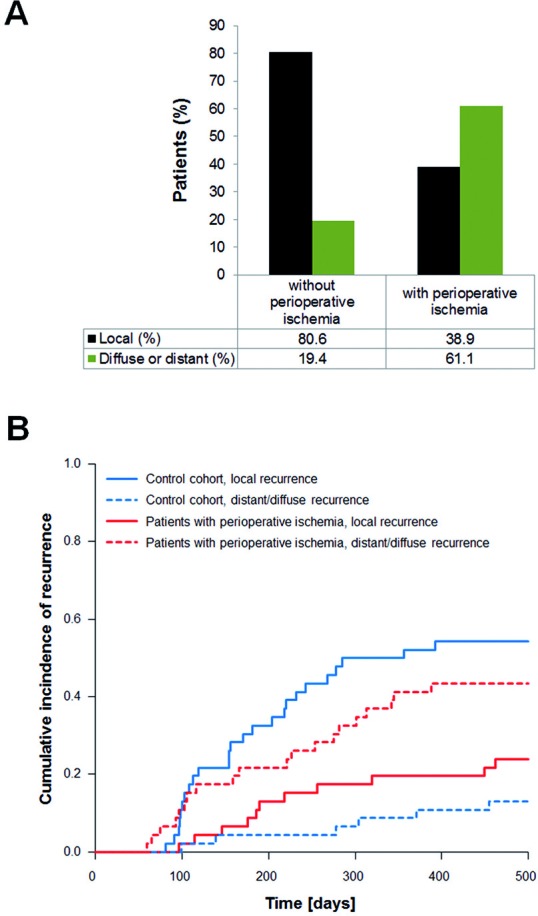
Frequency of patterns of progression **A**. shows the distribution of local and infiltrative recurrences (diffuse or distant) for patients with and without perioperative ischemia. **B**. shows the cumulative incidence of recurrences over time (competing risk analysis).

22 of 36 patients (61.1%) with perioperative ischemia showed an infiltrative pattern of progression (Figure 4A). In detail, diffuse recurrence was observed in 10 of 36 patients (27.8%) and distant recurrence in 12 of 36 patients (33.3%). Accordingly, local recurrence was only observed in 38.9% of the patients with perioperative ischemia (14 of 36 patients). In contrast, in the control cohort of matched pairs (patients without perioperative ischemia) only 19.4% (7 of 36 patients) showed an infiltrative pattern of progression (Figure 4A). Diffuse recurrence was observed in 2 of 36 patients (5.6%) and distant recurrence in 5 of 36 patients (13.9%). Local recurrence was observed in 80.6% of the control patients (29 of 36 patients). Notably, the results of the matched pair cohort correspond to previously published data on patterns of progression in patients with glioblastoma.[1, 18, 19]

### Competing risk analysis

To confirm that the difference between the two cohorts (*n* = 46 each) is significant we performed a competing risk analysis (Figure 4B). This analysis was done as described in Methods including drop-out patients. Taken together, patients with perioperative ischemia showed significantly more distant or diffuse recurrences than patients without ischemia (*p* = 0.0007). In contrast, patients of the control cohort (no ischemia) exhibited significantly more local recurrences (Figure 4B, *p* = 0.0011).

### Progression-free survival and overall survival

**Figure 5:**
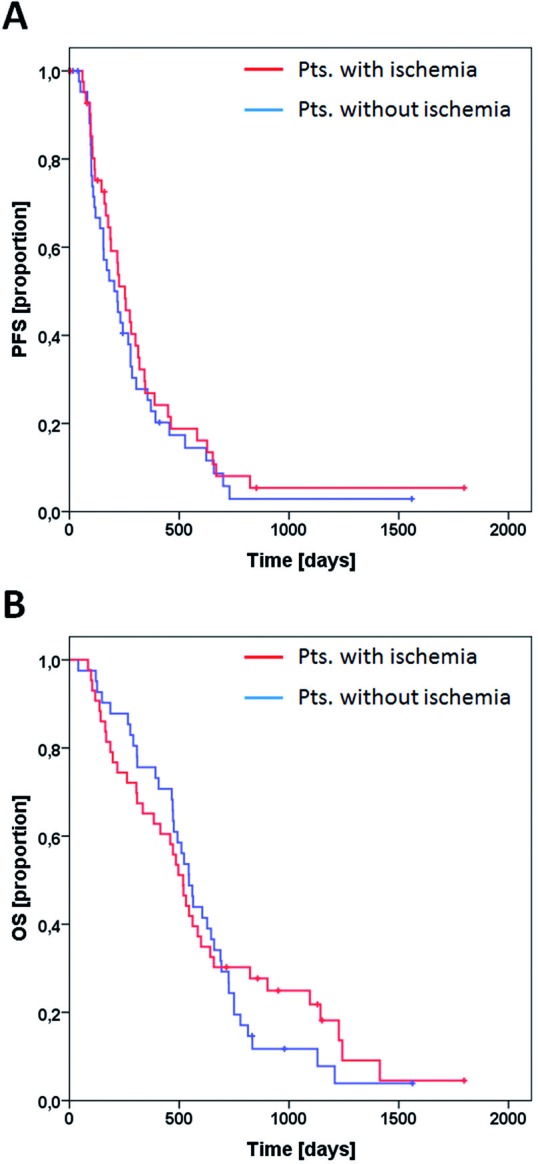
Survival analysis PFS for patients with perioperative ischemia (red line) and matched controls (blue line) are shown in **A**. OS for both groups is shown in **B**. Tick marks indicate censored patients.

Median time to progression was 253 days for patients with perioperative ischemia and 204 days for the control cohort (Figure 5A, *p* = 0.46). Median OS was 518 days for patients with perioperative ischemia and 544 days for the control cohort (Figure 5B, *p* = 0.65).

## DISCUSSION

This study reveals a previously unrecognized association of perioperative cerebral ischemia with distant or diffuse patterns of progression in patients with newly diagnosed glioblastoma.

The shift towards infiltrative patterns of progression in glioblastoma patients with relevant perioperative ischemia was substantial compared to the matched pair patient cohort and historical data.[1]

The percentage of diffuse or distant recurrences at first relapse was 19.4% in our control cohort and 61.1% in the cohort of patients with perioperative ischemia. Accordingly, the percentage of local recurrences dropped from 80.6% in the control cohort to 38.9% in the stroke cohort (Figure 4A). The distribution between local (80.6%) and infiltrative (19.4%) recurrences in our control cohort nicely matches with previous data. Chamberlain reported on 80% local relapses (64 of 80 patients) at first recurrence in glioblastoma patients.[1]

Interestingly, the increase in infiltrative growth in the ischemia group did not translate into worse progression free or overall survival (Figure 5).

Limitations of this study are (i) the small number of patients and (ii) the retrospective character. Nevertheless, the extent of the increase in infiltrative patterns of progression compared to our matched pair cohort and to historical data is impressive. The exact underlying mechanism for this increase cannot be clarified by this study. However, stroke is the best understood paradigm for cerebral ischemia. It appears likely that acute sublethal hypoxia during perioperative cerebral ischemia alters the tumor biology of affected residual glioma cells. Alternatively, cytokines released from ischemic tissue or systemic factors induced during cerebral ischemia may be involved. Hepatocyte growth factor (HGF) and Tissue Factor (TF) are two examples of a number of cytokines that show increased serum levels after cerebral ischemia and are known to induce glioma cell migration.[6, 20-25]

Our results match with findings in our previous study on BEV induced stroke-like lesions.[26] Patients that developed these lesions with restricted diffusion and T1 hyperintensities showed an increase of distant recurrences (41% vs. 18% in the cohort without stroke-like lesions). The histological evaluation showed extensive calcified necrosis in 4 out of 4 patients.[26] Furthermore, we found an increased HIF-1-alpha expression in an index patient with a lesion of restricted diffusion.[13, 14] We therefore hypothesized a true antiangiogenic effect with severe hypoxia leading to these stroke-like lesions and subsequent increase in distant relapses. Supporting this, preclinical and clinical studies have shown a BEV-induced increase in the expression of hypoxia inducible factor-1α (HIF-1α) or its target genes.[9-14]

In contrast, the majority of studies analyzing patterns of recurrence in glioblastoma patients receiving BEV have not found an increase in distant recurrences. However, these studies are based on low numbers of patients or have not discriminated responders from non-responders.[1, 18, 19]

There is no doubt that hypoxia is a key driver for infiltrative tumor growth in experimental glioma models. However, evidence that this is true in glioma patients is missing. In the current study we took advantage of the fact that some glioma patients suffer a cerebral ischemia during resection and thereby presumably exposing residual tumor cells to hypoxia. The magnitude of the increase of infiltrative tumor growth from approx. 20% to 60% is impressive. Beyond being the first clinical study demonstrating an effect of cerebral ischemia on tumor growth patterns our results are clinically relevant. Diffuse and distant relapses limit therapeutic options at recurrence and impair patients' performance status. Therefore, the cautiousness during resections is indispensable to avoid the adverse effect of perioperative ischemia. Further, this points out that hypoxia itself or the avoidance of hypoxia could serve as a reasonable therapeutic target to prevent the consequences of hypoxia on tumor biology. Two promising examples are hypoxia-activated drugs and oxygen delivery agents.

In summary, to the best of our knowledge, this is the first study on the influence of perioperative cerebral ischemia on patterns of progression in patients with newly diagnosed glioblastoma. Regarding the impact of hypoxia on tumor biology, this study supports the hypothesis that hypoxia induces infiltrative tumor growth in glioblastoma patients.

## MATERIALS AND METHODS

### Study population

We retrospectively screened postoperative MRI scans for perioperative ischemia in patients with newly diagnosed glioblastoma undergoing tumor resection between January 2008 and December 2012 (Figure 1). During that period 438 patients had first diagnosis of primary glioblastoma at our center. Out of this cohort 193 patients were biopsied and 245 patients underwent resection. Patients receiving bevacizumab (BEV) as part of their first line treatment and patients with small cerebral ischemia or cerebral ischemia distant from resection cavity were excluded (80 patients). Of the remaining 165 patients 46 showed new and relevant ischemia nearby the resection cavity on postoperative MRI (see below). Selection of control patients out of the remaining cohort of 119 patients without perioperative ischemia was done automatically by a software algorithm. The control cohort was generated by a 1:1 matched pairs design based on gender, age and adjuvant treatment. Both cohorts were analyzed for patterns of progression (local, diffuse or distant) by a blinded neuroradiologist. Further, we compared the results with historical data and estimated progression free and overall survival for both groups.

Our institutional review board approved this retrospective study and patients gave their consent for scientific work with clinical data including MRI scans (ethics committee at the University Hospital Frankfurt; reference number 4/09-SNO 01/08).

### Magnetic resonance imaging

Postoperative MRI was performed within 72 h following tumor resection on a 3 Tesla scanner (Siemens Medical AG). The protocol included T1-weighted (T1-w) sequences before and after intravenous administration of Gadolinium-containing contrast agent, T2-weighted sequences and DWI-sequences with calculated ADC-maps. To exclude postoperative hemorrhage as a cause of restricted diffusion T2*-weighted sequences were applied. DWI-Volume of each slice was approximated by multiplying the area of the ischemic lesions with the slice thickness including the gap between the slices. The volume of the whole ischemic lesion was calculated by adding the volumes of each slice. Only patients showing larger areas of relevant ischemia with high DWI-signal and corresponding low ADC-values nearby the resection cavity were enrolled (DWI-Volume > 3 cm³). MRI of a representative patient is shown in Figure 2. A small rim of diffusion restriction around the surgical cavity as often seen on postoperative MRI was not regarded as relevant ischemia. During follow-up the areas of restricted diffusion were assessed with particular attention. As expected, the course of these areas was typical for cerebral ischemia with temporary contrast enhancement and finally ischemic gliosis.

For follow-up all patients had T1-weighted (T1-w) sequences before and after intravenous administration of Gadolinium-containing contrast agent and T2-weighted sequences on a 1.5 Tesla scanner (Philips Medical Systems). Response was determined according to the updated response assessment criteria for high-grade gliomas (RANO).[27]

Patterns of progression were analyzed at first progression by one experienced neuroradiologist (M.W.) who was blinded for the scientific rationale of the analysis. We predefined modified categories as reported by Pope and colleagues and described previously.[18, 26]

Local: Enhancing or non-enhancing tumor at or within 3 cm of the primary site.

Diffuse: Recurrent tumor extending more than 3 cm from the primary site with at least 50% of the margin of the recurrent tumor poorly defined.

Distant: One or more new non-contiguous lesion (enhancing or not) with at least 1 cm distance to primary site.

Representative images for these three categories are shown in Figure 3. Diffuse and Distant recurrences were regarded as infiltrative patterns of progression and were combined for further analysis.

### Statistics

The generation of the matched pairs design was done with R Version 2.14 (R Foundation for Statistical Computing, Vienna, Austria) using the package Matching (generation of the matched pairs design).

Based on PFS the occurrence of the different types of recurrence (local and distant/diffuse) was analyzed with a competing risk analysis additionally accounting for the competing risks of death before progression, unknown type of progression (insufficient radiology) and censored patient that have not progressed yet. Competing risk analysis was done with R Version 2.14 (R Foundation for Statistical Computing, Vienna, Austria) using the package cmprsk (competing risk analysis).

Progression free survival (PFS) and overall survival (OS) from date of resection were estimated using the Kaplan-Meier method and analyzed via the Log-Rank-test (IBM SPSS Statistics Version 20.0). All tests were two-sided and p-values below 5% were considered significant.
